# Elevated versus irritable mood: is illness severity any different?

**DOI:** 10.1192/j.eurpsy.2023.469

**Published:** 2023-07-19

**Authors:** J. T. Coelho, A. S. Machado, F. Andrade, A. Vieira, S. Timóteo, A. Silva

**Affiliations:** Department of Psychiatry and Mental Health, University Hospital Center of São João, Porto, Portugal

## Abstract

**Introduction:**

Recent studies reported substantive clinical differences in those with a bipolar disorder who evidence elevated or irritable mood during a manic episode, which may have treatment and prognosis implications.

**Objectives:**

We aim to compare sociodemographic and clinical characteristics of inpatients admitted for bipolar mania with elevated vs. irritable mood.

**Methods:**

Retrospective observational study of inpatients admitted between January 1^st^ 2018 and July 31^st^ 2022 in a psychiatry inpatient unit of a tertiary hospital. Descriptive analysis of the results was performed using the SPSS software, version 26.0.

**Results:**

Our sample included 143 inpatients, 39,9% (n=57) with elevated mood. When compared with those with irritable mood, euphoric patients had 2.765 more odds of having previous psychiatric hospitalizations (*x*2(1, *N* = 143) = 4.93; *p* = 0.026). Interestingly, 78.4% of inaugural manic episodes (n=19) presented with irritable mood (*x*2(1, *N* = 143) = 3.447; *p* = 0.063). We also found that a patient with euphoric mood has 2.575 greater odds of being under a mood stabilizer (*x*2(1, *N* = 143) = 5.026; *p* = 0.025) before admission. More specifically, there is a significantly higher proportion of euphoric patients that were prescribed with valproic acid as mood stabilizer (57.9% vs 37.2%; *x*2(1, *N* = 143) = 5.016; *p* = 0.015). This association was not found with lithium. We found no statistically significant differences regarding the sociodemographic characteristics, previous long acting injectable antipsychotic or antidepressant treatment and psychotic symptoms during manic episode between the two groups.

**Conclusions:**

Patients with elevated mood are more likely to have a previous bipolar disorder diagnosis, which may reflect an observer bias due to the fact that diagnosis is already known.

The use of valproic acid as mood stabilizer may be a protective factor to irritable mood, since it’s currently prescribed in those with bipolar disorder who have more depressive or mixed instead of manic episodes. However, future studies are essential to understand the impact of mood stabilizer on these two contrasting phenotypic expressions.

Differences related to disease severity or sociodemographic characteristics were not found.

**Disclosure of Interest:**

None Declared

